# A short course of oral ranitidine as a novel treatment for toddler’s diarrhea: a parallel-group randomized controlled trial

**DOI:** 10.1186/s12887-020-02267-7

**Published:** 2020-08-11

**Authors:** Samuel N. Uwaezuoke, Ikenna K. Ndu, Chizoma I. Eneh, Chikere A. Anusiem, Adaeze C. Ayuk

**Affiliations:** 1 Department of Pediatrics, University of Nigeria of Nigeria Teaching Hospital Ituku-Ozalla Enugu/College of Medicine, University of Nigeria Enugu Campus, Enugu, Nigeria; 2 Department of Pediatrics, Enugu State University Teaching Hospital, Parklane, Enugu, Nigeria; 3 Department of Pharmacology and Therapeutics, College of Medicine, University of Nigeria Enugu Campus, Enugu, Nigeria; 4 Harvard University, Cambridge, USA

**Keywords:** Toddler’s diarrhea, Functional gastrointestinal disorders, Pharmacotherapy, Dietary modification, Probiotics, Inhibitors of gastric acid secretion

## Abstract

**Background:**

The current paradigm for treating toddler’s diarrhea comprises dietary modification and fluid restriction. Previous studies show that probiotics and proton-pump inhibitors (PPIs) or H_2_ blockers could control diarrhea associated with functional gastrointestinal disorders (FGIDs). This study aims to determine and compare the efficacy of a short course of oral ranitidine and a probiotic in the treatment of toddler’s diarrhea.

**Methods:**

This study was a parallel-group randomized controlled trial (RCT). We sequentially enrolled 40 patients who met the eligibility criteria. We randomly assigned 20 patients to the oral ranitidine group, ten patients to the probiotic group, and ten patients to the placebo group. In the oral ranitidine group, patients received oral ranitidine (3 mg/kg/day) once daily for 10 days; in the probiotic and placebo groups, they were administered 5 to 10 billion colony-forming units (CFUs) per day of lyophilized *Lactobacillus rhamnosus* and 50 mg of once-daily oral vitamin C tablet respectively for 10 days. Stool frequency and consistency on the 10th day of the interventions were recorded as the primary outcomes. We used the Student’s t-test to determine if there were significant differences in the mean daily stool frequencies in the three intervention groups. A *p*-value < 0.05 was adopted as the level of statistical significance.

**Results:**

In the ranitidine group, stool frequency decreased significantly from an average of five per day on the first day to an average of approximately one per day on the 10th day of intervention (t = 10.462, *p* <  0.001). Additionally, stool consistency normalized on the 10th day of intervention. In the probiotic group, there was a significant reduction in stool frequency from an average of five per day on the first day to four per day on the 10th day (t = 2.586, *p* = 0.041), although stool consistency remained loose. However, stool consistency and frequency were not significantly affected in the placebo group (t = 1.964, *p* = 0.072).

**Conclusion:**

Oral ranitidine is more effective than probiotics in reducing stool frequency and normalizing stool consistency in toddler’s diarrhea. We recommend multi-center trials with appropriate study designs to confirm and validate this finding.

**Trial registration:**

ISRCTN, ISRCTN10783996. Registered 8 April 2016-Registered retrospectively.

## Background

Toddler’s diarrhea or ‘chronic non-specific diarrhea of childhood’ is a common cause of persistent loose stools in under-five children [1, 2]. It refers to ‘chronic diarrhea lasting more than three weeks in a toddler who has normal anthropometric parameters’ coupled with the absence of fluid and electrolyte imbalance or systemic signs like vomiting and pyrexia [2]. The age bracket of affected children is between 6 and 40 months [3], or between 1 and 2 years [2]. Children with chronic non-specific diarrhea have a characteristic stooling pattern [2, 4]. The essential features include passage of foul-smelling, watery, loose, or ‘mushy’ stools (containing food remnants) during the day, which alternates with normal stool consistency and frequency at night.

Based on duration, diarrhea has been grouped into three types: short-duration watery diarrhea (lasting 7 to 14 days), persistent diarrhea (lasting 14 to 30 days), and chronic diarrhea (lasting more than 30 days). Based on pathophysiologic mechanisms, it has also been classified as secretory diarrhea (involves active secretion of chloride or inhibition of sodium and chloride absorption with concomitant fluid loss), osmotic diarrhea (non-absorbable substrates such as sorbitol or solutes draw fluid into the gut lumen), inflammatory diarrhea (damage to the gut-mucosal lining or brush border results in decreased absorptive capacity of lost protein-rich fluids) and functional diarrhea (no underlying structural or biochemical cause to explain the symptom).

Although toddler’s diarrhea was thought to be related to osmotic diarrhea, it is currently grouped among the functional gastrointestinal disorders (FGIDs) of childhood and has these synonyms: ‘functional diarrhea’ and ‘irritable colon of childhood’ [5]. Most authors however believe it is a gut motility disorder that is modulated by dietary factors and excessive fluid intake [1, 6–9]. Low-fat diets and the consumption of fruit juices, especially juices high in sorbitol and those with a high fructose: glucose ratio, have been strongly implicated [1, 9] Thus, dietary modification and reduction of daily fluid intake constitute the current paradigm of treatment. Other studies have shown that probiotics such as *Lactobacillus rhamnosus* and *Saccharomyces boulardii* could also resolve toddler’s diarrhea [10, 11]. Apart from its contra-indication in some categories of patients with immunosuppression, the use of probiotics (especially lyophilized *Lactobacillus rhamnosus* or *Saccharomyces boulardii*) in children is associated with minimal side effects: justifying their potential prescription in toddler’s diarrhea.

About two decades ago, Dave and Rubin reported that inhibition of gastric acid secretion with proton-pump inhibitors (PPIs) or H_2_ blockers effectively controlled the frequent bowel motion and post-prandial urgency associated with functional diarrhea or diarrhea-prominent irritable bowel syndrome (IBS-D) [12]. The authors attributed this novel finding to probable suppression of the gastro-colic or gastro-enteric reflex [12]. Their study can open a new vista for the use of these pharmacologic agents in treating toddler’s diarrhea. Ranitidine (an H_2_ blocker) is presently licensed for the treatment of peptic ulcers in children. Besides, its safety profile in children makes it an attractive therapeutic option for treating toddler’s diarrhea. Unlike cimetidine (an H_2_ blocker), which interferes with cytochrome P450 (CYP) pathway leading to impaired metabolism of several drugs through P450 enzymes [13], ranitidine is a less potent CYP inhibitor [14]. Also, famotidine (another H_2_ blocker) has a negligible effect on the CYP system with no significant drug interactions [13].

Given the pharmacokinetic property of ranitidine and the likelihood of inadequate parental compliance with dietary modification and fluid restriction, we sought to investigate the potential efficacy of this H_2_ blocker as an alternative pharmacologic therapy. We hypothesize that based on its action of suppressing the gastro-colic reflex, ranitidine can be a more effective treatment for toddler’s diarrhea. This randomized controlled trial (RCT) thus aims to determine and compare the efficacy of a short course of oral ranitidine and a probiotic in the treatment of toddler’s diarrhea. We conducted and reported this trial according to the CONSORT 2010 guidelines.

## Methods

### Trial design

The study was designed as parallel-group, single-blind, and explanatory RCT. It was a superiority trial in which ranitidine was hypothesized to be superior to probiotics in treating toddler’s diarrhea. Treatment allocation for each intervention group was achieved by using an allocation ratio of 2:1.

### Participants

The participants met the following eligibility criteria: age range of 12–36 months, duration of diarrhea lasting 3 weeks or more, historical evidence of the characteristic stooling pattern, absence of pyrexia and signs of dehydration, standard anthropometric measurements and normal findings on stool analysis and stool microscopy [2–4]. We conducted the trial at the Paediatric Out-patient Clinic, University of Nigeria Teaching Hospital (UNTH), located at Ituku-Ozalla (a satellite semi-urban area of Enugu metropolis), and a privately-run Pediatric Clinic (Restoration Medical Centre) located at Achara Layout within Enugu metropolis. However, the majority (30) of the trial participants were enrolled at the latter. We applied for and obtained ethical clearance and approval from the Health Research and Ethics Committee (HREC) of UNTH Ituku-Ozalla Enugu with the approval number: NHREC/05/01/2008B-FWA00002458-1RB00002323.

### Interventions

Participants’ enrolment, evaluation, and allocation to intervention groups were conducted between 02/05/2016 and 30/10/2019. We also evaluated their dietary history with an emphasis on artificial fruit-juice consumption, daily fluid intake, and consumption of the family menu. We used a structured proforma to document the following clinical characteristics of the participants: biodata, namely age and sex, anthropometric parameters consisting of weight, mid-upper arm circumference (MUAC) and height, findings of stool analysis and stool microscopy, as well as vital signs comprising axillary temperature (in degree centigrade), pulse rate and respiratory rate. In the oral ranitidine group, participants were given oral ranitidine (3 mg/kg/day) once daily for 10 days. In the probiotic group, we prescribed lyophilized *Lactobacillus rhamnosus* at 5 to 10 billion colony-forming units (CFUs) per day for the same duration. The participants in the placebo group received 50 mg daily of oral vitamin C (orange-flavored) tablets for 10 days. Parents received specific instructions on how they should administer the intervention medications at home.

### Outcomes

Stool frequency and consistency were the primary outcome measures. We extracted the information from the parents by telephone communication and recorded them on the 5th and 10th day of the three interventions using the structured proforma (which had specifications for data on stool frequency and consistency on days 1, 5, 10, and 30 of the intervention). We also inquired about any adverse drug reactions on these days. After the 10th day of trial, participants were followed up for documentation of their stool frequency and consistency up till the 60th day after each intervention through parental proxy reports on mobile telephony, as well as from scheduled clinic visits.

### Sample size determination

We used a predetermined table with the desired statistical power of 0.95 and a Cohen’s d (effect size) value of 0.8, which approximates to a sample size of 42. (The value of 0.8 was chosen because it corresponded to a ‘large’ effect size in the table, indicating the likelihood of a stronger effect). Two parents declined to participate so that 40 participants were randomly allocated as the final sample size.

### Randomization procedure

In the randomization procedure, we used the permuted-block randomization: adopting a block size of 30**.** Thus, we randomly assigned 20 participants to the oral ranitidine group and ten each to the probiotic and placebo groups based on our adopted allocation ratio of 2:1. We ensured allocation concealment by the use of sequentially numbered, opaque, sealed envelopes (SNOSE). The color-coded envelopes containing the medications were sequentially numbered irrespective of the color. Thus, the treatment to be allocated was unknown before each participant was entered into the trial. SNU generated the random allocation sequence, IKN enrolled the participants while CIE assigned the participants to the intervention groups.

### Blinding

After assignment to the intervention groups, each enrolled participant was given a color-coded, opaque envelope containing ranitidine, or probiotic or vitamin C. The parent was blinded in the trial; the content of each container was unknown to the parent and but was known to the investigators or outcome assessors.

### Statistical methods

We computed the frequencies and means of variables for the demographic data and clinical data of participants in the three intervention groups. We also compared the mean values of ages, anthropometric variables, and vital signs as well as the primary outcome measures in the groups using the Duncan multiple comparison test. We used the Student’s t-test to determine if there were any differences in their mean daily stool frequencies on the 10th day of the three interventions. For its estimation in absolute terms, we used the ranitidine and probiotic groups for reporting the effect size or effect estimate (EE). We employed two standardized measures: Cohen’s d and estimation of absolute risk reduction or risk difference (RD). A 95% confidence interval (CI) was assumed. A *p*-value < 0.05 was adopted as the level of statistical significance.

## Results

### Participants’ flow diagram

**Fig. 1:**
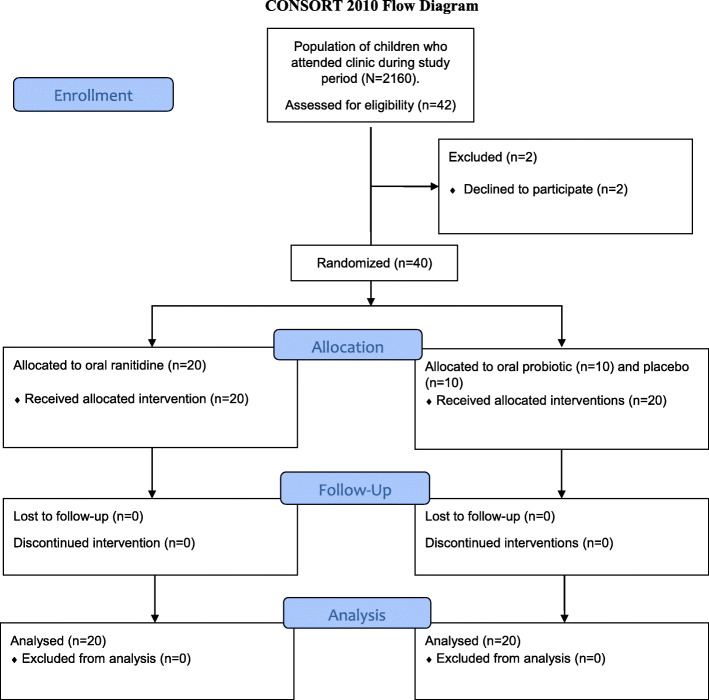
Participants’ flow diagram showing their assignment, the interventions and analysis

As shown in Fig. 1 (CONSORT 2010 Flow Diagram), 42 subjects who met the eligibility criteria were selected from an average population of 2160 children seen in the clinic over the study period. After two parents declined to participate in the trial, 40 participants were randomly assigned to the three intervention groups: 20 to oral ranitidine group, 10 to the probiotic group, and 10 to the placebo group. All (40 participants) received the intended interventions and were subsequently analyzed for stool frequency per day and stool consistency on the 10th day of treatment. The periods of recruitment and follow-up were between 02/05/2016 and 30/10/2019. The trial ended when the assignment and analysis of the target sample size of 40 participants were achieved.

### Baseline data

Table 1 shows the demographic data and clinical characteristics of the participants. The mean age values (in months) for participants in the ranitidine, probiotic and placebo groups were 23.90 ± 9.41, 17.70 ± 7.62, and 17.7 ± 4.11 respectively; these were not significantly different (*p* = 0.061). Their mean weights were 12.85 ± 2.89 kg (ranitidine group), 11.40 ± 1.37 kg (probiotic group) and 13.30 ± 2.88 kg (placebo group); their mean heights, 90.73 ± 7.35 cm (ranitidine group), 86.30 ± 4.22 cm (probiotic group) and 85.70 ± 2.11 cm (placebo group); and their mean mid-upper arm circumference, 15.25 ± 0.72 cm (ranitidine group), 15.10 ± 0.84 cm (probiotic group) and 15.70 ± 0.92 cm (placebo group). The calculated mean weight-for-age (W/A) for ranitidine group and for both probiotic and placebo groups were 11.98 kg and 10.95 kg respectively, while the mean height-for-age (H/A) estimates were 88.95 cm for ranitidine group, and 85.85 cm for probiotic and placebo groups. Additionally, the mean weight-for-height (W/H) Z scores for ranitidine, probiotic and placebo groups were 0.013, 0.001 and 0.003 respectively. Whereas the difference in the mean heights of the three intervention groups was significant (*p* = 0.045), the differences in their mean weights (*p* = 0.233) and mid-upper arm circumference (*p* = 0.220) were not significant. Regarding vital signs, the mean pulse rates for the intervention groups were 102.40 ± 4.03 per minute (ranitidine group), 104.00 ± 3.13 per minute (probiotic group) and 103.80 ± 2.39 per minute (placebo group). Their mean temperature values were 36.92 ± 0.23 ^o^ Centigrade (ranitidine group), 36.82 ± 0.23 ^o^ Centigrade (probiotic group) and 36.92 ± 0.31 ^o^ Centigrade (placebo group) while their mean respiratory rates were 31.50 ± 3.36 per minute (ranitidine group), 32.80 ± 2.53 per minute (probiotic group) and 34.60 ± 1.35 per minute (placebo group). The mean respiratory rate values of the groups varied significantly (*p* = 0.024) while their mean temperature values (*p* = 0.561) and mean pulse rates (0.401) were not significantly different.

**Table 1:** Comparison of mean values of ages and clinical characteristics of participants in the three intervention groups

Variables	Oral ranitidine ^d^ Mean ± SD	Probiotic ^b d^ Mean ± SD	Placebo^c d^ Mean ± SD	F	*p*-value^**†**^
Age (months)	23.90 ± 9.41	17.70 ± 7.62	17.70 ± 4.11	3.016	0.061
Male/Female ratio	13/7	6/4	4/6		
Weight (kilogram)	12.85 ± 2.89	11.40 ± 1.37	13.30 ± 2.88	1.517	0.233
MUAC (centimeter)	15.25 ± 0.72	15.10 ± 0.84	15.70 ± 0.92	1.577	0.220
Height (centimeter)	90.73 ± 7.35	*86.30 ± 4.22	*85.70 ± 2.21	3.385	0.045
Pulse rate	102.40 ± 4.03	104.00 ± 3.13	103.80 ± 2.39	0.937	0.401
Temperature^a^	36.92 ± 0.23	36.82 ± 0.23	36.92 ± 0.31	0.587	0.561
Respiratory rate	*31.50 ± 3.36	*32.80 ± 2.53	34.60 ± 1.35	4.135	0.024

*Duncan multiple comparison tests indicating means not significantly different. ^**†**^*p* value < 0.05 adopted as statistically significant

^a^Measured in degree centigrade ^b^ Lyophilized lactic acid bacteria ^c^ Vitamin C tablet

*SD* standard deviation, *MUAC* mid-upper arm circumference

^d^Mean weight-for-age (W/A) for ranitidine, probiotic, and placebo groups were 11.98 kg, 10.95 kg and 10.95 kg respectively. Mean height-for-age (H/A) were 88.95 cm (ranitidine group), 85.85 cm (probiotic and placebo groups). Mean weight-for-height (W/H) Z scores were 0.013 for ranitidine group, 0.001 for probiotic group and 0.003 for placebo group

Of the 40 participants analyzed, 23 (57.5%) were males, while 17 (42.5%) were females (Table 1). There were more males than females in the oral ranitidine group (*n* = 13 [56.5%] versus *n* = 6 [41.2%]) and the probiotic group (*n* = 6 [26.1%] versus *n* = 4 [23.5%]). However, there were more females than males in the placebo group (*n* = 6 [35.3%] versus *n* = 4 [17.4%]). These differences were not statistically significant (*p* = 0.419).

### Outcomes and estimations

**Fig. 2:**
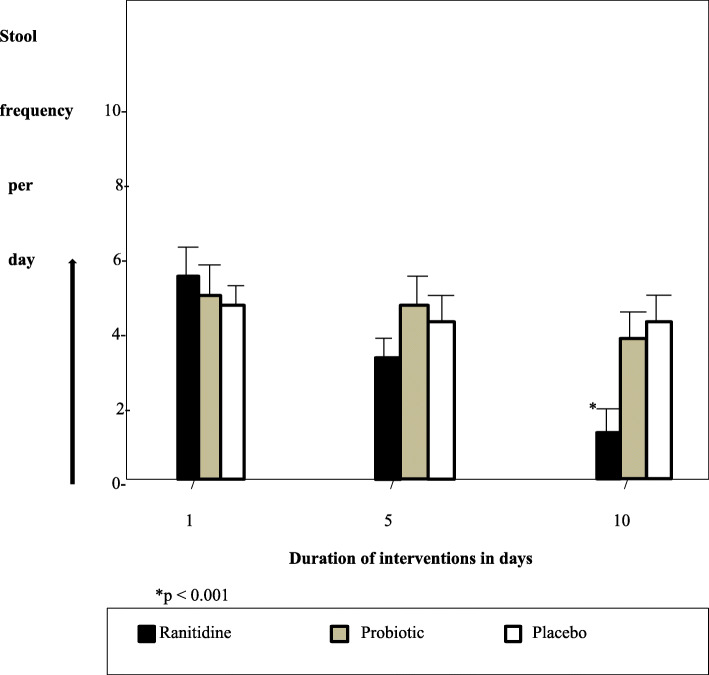
Bar graphs showing the effect of the three interventions on average daily stool frequency within 10 days

The primary outcomes were stool frequency per day and stool consistency. The pre-intervention mean stool frequencies per day for each group were 5.35 ± 1.76 (ranitidine group), 5.30 ± 1.42 (probiotic group) and 5.30 ± 1.06 (placebo group). On the 10th day of the interventions, the mean stool frequencies per day were 1.30 ± 0.47 (ranitidine group), 4.40 ± 0.84 (probiotic group) and 5.00 ± 0.94 (placebo group). The change in the mean daily stool frequency for the ranitidine group (t = 10.462, *p* <  0.001) and the probiotic group (t = 2.586, *p* = 0.041) were statistically significant. There was no significant change in the placebo group (t = 1.964, *p* = 0.072). As shown in Fig. 2, a progressive reduction in the average daily stool frequency occurred in the 5th and 10th day of intervention with oral ranitidine. The value decreased significantly from an average of five per day to approximately one per day on the 10th day. In Table 2, a comparison of the mean daily stool frequency and stool consistency among the three intervention groups shows a statistically significant difference in the former on the 10th day (F = 116.769, *p* < 0.001). Stool consistency was formed in the ranitidine group but remained loose in the probiotic and placebo groups.

**Table 2:** Comparison of mean stool frequency and stool consistency on the 10th day of various interventions

Primary outcomes	Oral ranitidine^a^ Mean ± SD	Probiotic^b^ Mean ± SD	Placebo^c^ Mean ± SD	F	*p-*value*
Stool frequency	1.30 ± 0.47	4.40 ± 0.84	5.00 ± 0.94	116.769	< 0.001
Stool consistency	Formed	Loose	Loose		

*SD* standard deviation *p-value < 0.05 adopted as statistically significant

^a^3 mg/kg of daily oral ranitidine

^b^5 to 10 billion colony-forming units per day of lyophilized lactic acid bacillus

^c^50 mg of daily vitamin C tablet

The measured effect size or effect estimate (EE) using Cohen’s d standardized method (based on the mean daily stool frequencies for the ranitidine and placebo groups on the 10th day) was 2.62. Based on the mean daily stool frequencies for the ranitidine and probiotic groups on the 10th day, the EE was 2.36. However, based on the mean daily stool frequencies for the probiotic and placebo groups on the 10th day, EE was 0.34. Thus, the small sample size used in this trial appeared appropriate to validate the effect of ranitidine, while a larger sample size would rather be required to establish the efficacy of probiotics. Comparing the ranitidine and placebo groups, the estimated absolute risk reduction or RD effect size concerning mean daily stool frequency was 0.29 (29%). In contrast, the RD effect size in comparing the probiotic and placebo groups regarding the same primary outcome was 0.04 (4%): both values based on the assumption of 95% CI.

No adverse drug reactions were reported in the participants in the three groups throughout the trial.

## Discussion

In the present study, we noted that a daily dose of oral ranitidine effectively resolved the symptoms of toddler’s diarrhea on the tenth day of treatment. A substantial reduction in daily stool frequency and improvement in stool consistency had also occurred by the fifth day of therapy. Patients remained symptom-free 60 days after the stoppage of the intervention. These observations were similar to the findings reported by Dave and Rubin [12]. They studied twenty patients with chronic diarrhea and postprandial urgency due to IBS-D or functional diarrhea, of whom fourteen patients received PPIs, and six patients, H_2_ blockers. Remarkably, they observed rapid symptom-resolution within 3 days as their patients had one to three formed stools per day. More importantly, patients were followed up on therapy, and symptom resolution was sustained for one to 6 months [12]. The authors investigated the treatment of FGIDs with these inhibitors of gastric acid secretion. According to them, it was prompted by anecdotal evidence of rapid and unexpected relief of chronic functional diarrhea and postprandial urgency in five of their patients with gastroesophageal reflux disease (GERD). These patients received a PPI (lansoprazole) for heartburn. Whereas their study was observational, our work was an RCT that compared the efficacy of two pharmacologic agents with placebo as the control.

The current perspectives on the treatment of childhood FGIDs show that the majority of them, including toddler’s diarrhea, is primarily managed with non-pharmacologic methods [15, 16]. As previously mentioned, the current modality of treating toddler’s diarrhea comprises dietary modification and fluid restriction [1]. Over the years, the efficacy of other pharmacologic agents in treating toddler’s diarrhea and IBS-D have been investigated [12, 17–24]. Although the use of PPIs and H_2_ blockers were found promising [12], drugs like diodoquin, aspirin, loperamide, bile-acid sequestrant (cholestyramine), serotonin receptor antagonists (alosetron, ramosetron, and ondansetron) and absorbents (diosmectite) were associated with minimal benefits [17–24]. Unfortunately, most of them resulted in adverse reactions, leading to their discontinuation.

We noted by anecdotal evidence that ranitidine could also effectively resolve postprandial urgency and stooling in children. For all these findings, the mechanism of action for the efficacy of PPIs or H_2_ blockers in IBS-D and toddler’s diarrhea appears to be the suppression of the gastro-colic reflex, as previously proposed [12]. This physiological reflex modulates the postprandial peristalsis of the gastrointestinal tract. Neuropeptide mediators of the gastro-colic reflex include serotonin, neurotensin, cholecystokinin, prostaglandin E1, and gastrin [25]. Gastrin is a peptide hormone that triggers the release of gastric acid and helps in gastric motility. If these dual functions of gastrin are inextricably linked, it is not surprising that inhibitors of gastric acid secretion would ultimately suppress the gastro-colic reflex and reduce intestinal transit time. Based on the reported benefits of bile-acid sequestrant (cholestyramine) and serotonin antagonists in IBS-D, antagonism of the mediators of the gastro-colic reflex may contribute to their therapeutic effect. Besides H_2_ blockers may delay gastric emptying, and their prolonged use may suppress gastric acid and thus promote intragastric colonization of pathogenic bacteria. Worse still, H_2_ blockers may interfere with the absorption of micronutrients such as iron and vitamins. These adverse effects strongly justify the short-duration course of ranitidine used in this clinical trial.

Secondly, we observed that the use of a probiotic (lyophilized *Lactobacillus rhamnosus*) was not effective in completely resolving the symptoms of toddler’s diarrhea. For instance, the average daily stool frequency decreased from five per day on the first day to four per day on the 10th day of treatment, with no change in stool consistency. Although the efficacy of probiotics has been previously reported in toddler’s diarrhea [11], a recent meta-analysis shows that only a few clinical studies documented its benefits in IBS-D [26]. In contrast, most studies did not indicate improvements in stool frequency and consistency associated with IBS-D [26]. We may not be able to correctly advance the reason for the inadequate response seen in our patients. However, it may be related to the strain or dosage of the probiotic used for this trial. Previous observations show that the potential efficacy of probiotics to treat antibiotic-associated diarrhea depends on the probiotic strains and dosage [27, 28]. In children, *Lactobacillus rhamnosus* and *Saccharomyces boulardii* were particularly recommended at 5 to 40 billion CFUs per day for preventing antibiotic-associated diarrhea [29], and are useful in treating persistent diarrhea as well [30]. Also, *Lactobacillus* strains were most effective in treating acute diarrhea at more than 10 billion CFUs during the first 2 days of diarrhea [31]. Thus, it appears that the efficacy of probiotics in resolving these forms of diarrhea is dose-dependent [32]. Given that we used a dosage that did not exceed 10 billion CFUs per day in our patients, we speculate that this may explain their inadequate response to the probiotic. Since probiotics improve the symptoms of IBS-D through their influence on intestinal transit time [26], one would have expected a similar efficacy in toddler’s diarrhea (another form of FGIDs).

Nevertheless, one report shows that probiotics may be beneficial in toddler’s diarrhea [11] and other forms of diarrhea, such as traveler’s diarrhea, antibiotic-associated diarrhea, and IBS-D [33, 34]. A meta-analytical study of blinded, randomized, and placebo-controlled trials also indicated that probiotics significantly decreased the symptoms of acute diarrhea [35]. Although probiotics appear to stabilize a dysfunctional gut, the exact mechanism of action in resolving diarrhea associated with FGIDs remains unclear. Some authors, however, suggest that they aid in the expression of the SLC26A3 gene, which initiates the production of the CLD-chloride anion exchanger involved in the modulation of ion absorption and secretion [36]. This mechanism particularly applies to antibiotic-induced diarrhea. In IBS-D, probiotics presumably improve disease symptoms through the preservation of gut microbiota, improvement in the intestinal transit time, and reducing small intestinal permeability [26].

The strength of our trial lies in its potential external validity. Concerning the effect of ranitidine, we presume our findings can be validly generalized, given the presumed adequate sample size. Our measured EE of 2.62 supports our small sample size, which lends credence to the trial efficacy of ranitidine. Better still, the calculated absolute risk reduction or RD of 29% when compared to RD of 4% for probiotic further underscores the effectiveness of oral ranitidine in toddler’s diarrhea.

On the other hand, our trial has some limitations. Firstly, the small sample size of the study precludes making a definite conclusion about the effect of the probiotic. With the EE e of 0.34 and the RD of 4%, a larger sample size would be required to establish its actual efficacy in toddler’s diarrhea. Secondly, we failed to consider confounders like food allergies and previous antibiotic use during the enrollment of the trial participants. We could only establish a brief dietary history that focused on the participants’ artificial fruit juice consumption and daily water intake. Food allergies and antibiotic use are associated with diarrhea. Besides, probiotics are known to affect diarrhea related to these confounding factors [29]. Thirdly, some demerits of probiotics could have affected the findings of our trial. These include a dearth of evidence about the strain-specific effects, poor standardization for clinical trial designs, insufficient quality of some brands, and variations in the microbial preparations [37]. Fourthly, evaluating the primary outcome measures was subjective as we depended on telephone communication with the parents to extract the information without directly observing these parameters, although there were scheduled clinic follow-ups up till the 60th day. However, the same subjectivity was evident at the stage of enrollment when we had to rely on the historical account of the parents to establish the initial stool frequency per day. Finally, we did not follow up on our patients beyond 60 days after the stoppage of intervention with oral ranitidine to determine the long-term effect of this treatment. We, however, presume that recurrence was rare since there were no self-reports by the parents of participants several months after the intervention. Nevertheless, a long-term follow up is required to determine disease recurrence with the short-course of this drug.

## Conclusions

H_2_ blockers like ranitidine may represent a paradigm shift in the treatment of toddler’s diarrhea. Like PPIs, which are associated with few side-effects, ranitidine is safe in children. Although toddler’s diarrhea is benign and self-limiting, there may be difficulty in parental compliance with the traditional dietary modification and fluid restriction. Moreover, the frequent recourse to antibiotic use in treating diarrheal diseases in the developing world underscores the need for a safe and effective therapeutic agent whose prescription will elicit better compliance. Compared with probiotics, a short course of oral ranitidine appears more effective in reducing stool frequency in toddler’s diarrhea. Besides, the use of probiotics may be complicated by bacteremia in immunocompromised patients and are thus associated with possible fatal complications. We, therefore, recommend multi-center trials with appropriate study designs to confirm and validate this finding.

## Supplementary information

**Additional file 1.**

**Additional file 2.**

**Additional file 3.**

## Data Availability

All data generated or analyzed during this study are found in the ISRTCN registry (ISRCTN10783996) and presented as supplementary file.

## Abbreviations

CFUs: Colony-forming units
CI: Confidence interval
CYP: Cytochrome P450
EE: Effect estimate
FGIDs: Functional gastrointestinal disorders
GERD: Gastroesophageal reflux disease
IBS-D: Diarrhea-prominent irritable bowel syndrome
MUAC: Mid-upper arm circumference
PPIs: Proton-pump inhibitors
RCT: Randomized controlled trial
RD: Risk difference
SNOSE: Sequentially numbered opaque sealed envelopes
