# Supplementary material for: A short course of oral ranitidine as a novel treatment for toddler’s diarrhea: a parallel-group randomized controlled trial
Source: BMC Pediatr. 2020 Aug 11;20:380. doi: 10.1186/s12887-020-02267-7 (PMC7422520; doi:10.1186/s12887-020-02267-7)
Supplement: Supplementary file 1 — Additional file 1. [file 12887_2020_2267_MOESM1_ESM.docx]

**Appendix 1**

**PARENTS’ OR CARE-GIVERS’ CONSENT FORM**

This study aims to determine the efficacy of the drug- ranitidine- in treating toddler’s diarrhoea (which your child is presenting with).

The nature of the interventions will be duly explained to you. We wish to enroll your child to any of the intervention groups: **oral ranitidine**, **oral probiotic** and **placebo (vitamin C) groups**

Thank you.

**Study centre investigator** (on behalf of co-investigators)

I …………………………… hereby consent to the enrolment of my child into the study after due explanation to me about the nature of the study and the interventions. Any adverse outcome of the medication to be given to my child has also been explained to me.

**Name………………………………………………… Signed**…………………………………

**Date**……………………………………..
