# Supplementary material for: A short course of oral ranitidine as a novel treatment for toddler’s diarrhea: a parallel-group randomized controlled trial
Source: BMC Pediatr. 2020 Aug 11;20:380. doi: 10.1186/s12887-020-02267-7 (PMC7422520; doi:10.1186/s12887-020-02267-7)
Supplement: Supplementary file 2 — Additional file 2. [file 12887_2020_2267_MOESM2_ESM.docx]

**ISRCTN10783996**

**RE: Oral ranitidine is effective in treating toddler’s diarrhea**

1. **Participants’ Flow: enrolment age – 1 to 3 years (12 to 36 months)**

Enrolment (n=40)

Oral ranitidine group (n=20)

Intervention allocation

(n=40) Placebo (vitamin C) group (n=10)

Probiotic (*lactobacillus*) group (n=10)

Follow-up (n=40)

Data analysis (n=40)

1. **Baseline characteristics of participants (Oral ranitidine group) - at enrolment**

| **Serial number of patients** | **Age**  **(months)** | **Gender** | **Weight**  **(kg)** | **MUAC^†^**  **(cm)** | **Height**  **(cm)** | **Pulse**  **rate^‡^** | **Temperature**  **(^o^C)** | **Respiratory**  **rate^§^** | **Dehydration** |
| --- | --- | --- | --- | --- | --- | --- | --- | --- | --- |
| 1. | 36 | Male | 15 | 16 | 96 | 102 | 37.2 | 30 | Nil |
| 2. | 33 | Male | 14 | 16 | 100 | 106 | 37.2 | 32 | Nil |
| 3. | 12 | Male | 10.5 | 14.5 | 80 | 108 | 36.8 | 36 | Nil |
| 4. | 20 | Female | 11 | 16 | 98 | 104 | 36.7 | 32 | Nil |
| 5. | 35 | Male | 13.5 | 15 | 90.5 | 98 | 36.8 | 24 | Nil |
| 6. | 26 | Male | 14 | 15 | 88 | 110 | 37 | 28 | Nil |
| 7. | 16 | Female | 10 | 15 | 84 | 102 | 36.9 | 30 | Nil |
| 8. | 36 | Male | 13 | 14.5 | 89 | 104 | 36.8 | 28 | Nil |
| 9. | 20 | Male | 13 | 15 | 88 | 102 | 37.1 | 36 | Nil |
| 10. | 15 | Female | 10 | 14 | 86 | 98 | 36.8 | 28 | Nil |
| 11. | 16 | Male | 10 | 14 | 86 | 102 | 36.5 | 36 | Nil |
| 12. | 24 | Female | 13 | 15 | 89 | 104 | 36.9 | 34 | Nil |
| 13. | 12 | Female | 10 | 16 | 83 | 106 | 37.3 | 32 | Nil |
| 14. | 19 | Male | 11 | 15.5 | 85 | 102 | 36.6 | 34 | Nil |
| 15. | 36 | Male | 18 | 16 | 94 | 96 | 36.9 | 30 | Nil |
| 16. | 34 | Male | 21 | 16 | 106 | 98 | 36.7 | 28 | Nil |
| 17. | 36 | Male | 15 | 16 | 98 | 104 | 37.2 | 32 | Nil |
| 18. | 26 | Female | 13 | 15 | 104 | 94 | 36.8 | 30 | Nil |
| 19. | 14 | Female | 12 | 16 | 84 | 106 | 37.3 | 36 | Nil |
| 20. | 12 | Male | 10 | 14.5 | 86 | 102 | 36.9 | 34 | Nil |

**^†^MUAC, mid upper arm circumference. ^‡^in beats/minute. ^§^in breaths/minute**

1. **Baseline characteristics of participants (probiotic group) – at enrolment**

| **Serial number**  **of patients** | **Age (months)** | **Gender** | **Weight**  **(kg)** | **MUAC^†^**  **(cm)** | **Height (cm)** | **Pulse**  **rate^‡^** | **Temperature**  **(^o^C)** | **Respiratory**  **rate^§^** | **Dehydration** |
| --- | --- | --- | --- | --- | --- | --- | --- | --- | --- |
| 1. | 15 | Male | 12 | 15 | 87 | 108 | 37.1 | 36 | Nil |
| 2. | 12 | Female | 10.5 | 14 | 84 | 102 | 36.9 | 34 | Nil |
| 3. | 36 | Male | 14 | 16 | 95 | 98 | 36.6 | 28 | Nil |
| 4. | 18 | Female | 11 | 16 | 89 | 104 | 37.2 | 34 | Nil |
| 5. | 13 | Male | 10 | 14.5 | 80 | 108 | 36.8 | 36 | Nil |
| 6. | 12 | Male | 10 | 15 | 85 | 106 | 36.5 | 32 | Nil |
| 7. | 24 | Female | 13 | 16 | 90 | 102 | 36.8 | 30 | Nil |
| 8. | 11 | Male | 10 | 14 | 84 | 106 | 36.7 | 32 | Nil |
| 9. | 16 | Female | 12 | 16 | 86 | 104 | 36.6 | 34 | Nil |
| 10. | 20 | Male | 11.5 | 14.5 | 83 | 102 | 37 | 32 | Nil |

**^†^MUAC, mid upper arm circumference. ^‡^in beats/minute. ^§^in breaths/minute**

1. **Baseline characteristics of participants (placebo -vitamin C) group – at enrolment**

| **Serial number**  **of patients** | **Age (months)** | **Gender** | **Weight**  **(kg)** | **MUAC^†^**  **(cm)** | **Height (cm)** | **Pulse**  **rate^‡^** | **Temperature**  **(^o^C)** | **Respiratory**  **rate^§^** | **Dehydration** |
| --- | --- | --- | --- | --- | --- | --- | --- | --- | --- |
| 1. | 12 | Female | 10.5 | 15 | 84 | 104 | 36.9 | 34 | Nil |
| 2. | 20 | Female | 11 | 14 | 85 | 104 | 37.3 | 36 | Nil |
| 3. | 13 | Male | 11.5 | 16 | 88 | 102 | 36.6 | 34 | Nil |
| 4. | 15 | Female | 12 | 15.5 | 86 | 106 | 37.3 | 34 | Nil |
| 5. | 24 | Male | 14 | 16 | 89 | 100 | 37.1 | 36 | Nil |
| 6. | 14 | Male | 12 | 16 | 83 | 108 | 36.8 | 34 | Nil |
| 7. | 19 | Female | 16 | 16 | 84 | 104 | 36.5 | 36 | Nil |
| 8. | 19 | Female | 20 | 17.5 | 85 | 102 | 36.6 | 32 | Nil |
| 9. | 23 | Female | 14 | 16 | 89 | 102 | 36.8 | 34 | Nil |
| 10. | 18 | Male | 12 | 15 | 84 | 106 | 37.3 | 36 | Nil |

**^†^MUAC, mid upper arm circumference. ^‡^in beats/minute. ^§^in breaths/minute**

1. **Study outcome measures (primary) - oral ranitidine group**

| **Serial number of patients** | **Pre-intervention stool consistency/frequency** | **Stool consistency/frequency*** | **Stool consistency/frequency†** | **Post-intervention**  **stool consistency/frequency‡** | **Post-intervention stool consistency /frequency§** |
| --- | --- | --- | --- | --- | --- |
| 1. | Loose/6 per day | Loose/5 per day | Formed/2 per day | Formed/1 per day | Formed/1 per day |
| 2. | Loose/10 per day | Loose/8 per day | Formed/1 per day | Formed/1 per day | Formed/1 per day |
| 3. | Loose/5 per day | Loose/5 per day | Formed/1 per day | Formed/1 per day | Formed/1 per day |
| 4. | Loose/4 per day | Loose/4 per day | Formed/1 per day | Formed/1 per day | Formed/1 per day |
| 5. | Loose/5 per day | Loose/4 per day | Formed/1 per day | Formed/1 per day | Formed/1 per day |
| 6. | Loose/5 per day | Loose/4 per day | Formed/1 per day | Formed/2 per day | Formed/1 per day |
| 7. | Loose/4 per day | Loose/4 per day | Formed/1 per day | Formed/1 per day | Formed/1 per day |
| 8. | Loose/3 per day | Loose/3 per day | Formed/1 per day | Formed/1 per day | Formed/1 per day |
| 9. | Loose/4 per day | Loose/3 per day | Formed/2 per day | Formed/1 per day | Formed/1 per day |
| 10. | Loose/4 per day | Loose/3 per day | Formed/1 per day | Formed/1 per day | Formed/1 per day |
| 11. | Loose/4 per day | Loose/4 per day | Formed/1 per day | Formed/1 per day | Formed/I per day |
| 12. | Loose/5 per day | Loose/3 per day | Formed/1 per day | Formed/1 per day | Formed/1 per day |
| 13. | Loose/ 6 per day | Loose/4 per day | Formed/2 per day | Formed/1 per day | Formed/1 per day |
| 14. | Loose/ 8 per day | Loose/6 per day | Formed 2 per day | Formed/1 per day | Formed/2 per day |
| 15. | Loose/4 per day | Loose/4 per day | Formed/1 per day | Formed/1 per day | Formed/ 1 per day |
| 16. | Loose/ 5 per day | Loose/4 per day | Formed/2 per day | Formed/1 per day | Formed/1 per day |
| 17. | Loose/7 per day | Loose/5 per day | Formed/1 per day | Formed/1 per day | Formed/1 per day |
| 18. | Loose/6 per day | Loose/4 per day | Formed/2 per day | Formed/1 per day | Formed/1 per day |
| 19. | Loose/8 per day | Loose/6 per day | Formed/1 per day | Formed/2 per day | Formed/1 per day |
| 20. | Loose/4 per day | Loose/3 per day | Formed/1 per day | Formed/1 per day | Formed/1 per day |

***5^th^ day of intervention. †10^th^ day of intervention. ‡30 days after stopping intervention. § 60 days after stopping intervention**

1. **Study outcome measures (primary) – probiotic group**

| **Serial number of patients** | **Pre-intervention stool consistency/ frequency** | **Stool consistency/frequency*** | **Stool consistency/ frequency†** | **Switch- over to oral ranitidine** | **Stool consistency/frequency†** | **Post-intervention stool consistency/frequency‡** | **Post-intervention stool consistency/**  **frequency§** |
| --- | --- | --- | --- | --- | --- | --- | --- |
| 1. | Loose/4 per day | Loose/4 per day | Loose/4 per day | Loose/4 per day | Formed/1 per day | Formed/1 per day | Formed/1 per day |
| 2. | Loose/5 per day | Loose/4 per day | Loose/4 per day | Loose/4 per day | Formed/1 per day | Formed/1 per day | Formed/1 per day |
| 3. | Loose/4 per day | Loose/4 per day | Loose/4 per day | Loose/4 per day | Formed/1 per day | Formed/1 per day | Formed/1 per day |
| 4. | Loose/5 per day | Loose/5 per day | Loose/5 per day | Loose/5 per day | Formed/1 per day | Formed/1 per day | Formed/1 per day |
| 5. | Loose/6 per day | Loose/6 per day | Loose/6 per day | Loose/6 per day | Formed/2 per day | Formed/1 per day | Formed/1 per day |
| 6. | Loose/8 per day | Loose/6 per day | Loose/5 per day | Loose/5 per day | Formed/1 per day | Formed/1 per day | Formed/1 per day |
| 7. | Loose/4 per day | Loose/3 per day | Loose/3 per day | Loose/3 per day | Formed/1 per day | Formed/1 per day | Formed/1 per day |
| 8. | Loose/6 per day | Loose/4 per day | Loose/4 per day | Loose/4 per day | Formed/1 per day | Formed/1 per day | Formed/1 per day |
| 9. | Loose/4 per day | Loose/4 per day | Loose/4 per day | Loose/4 per day | Formed/1 per day | Formed/1 per day | Formed/1 per day |
| 10. | Loose/7 per day | Loose/5 per day | Loose/5 per day | Loose/5 per day | Formed/2 per day | Formed/1 per day | Formed/1 per day |

***5^th^ day of intervention. †10^th^ day of intervention. ‡30 days after stopping intervention with oral ranitidine. § 60 days after stopping intervention**

**Study outcome measures (primary) - placebo (vitamin C) group**

| **Serial number of patients** | **Pre-intervention stool consistency/frequency** | **Stool consistency/ frequency^*^** | **Stool consistency/frequency^†^** | **Switch- over to oral ranitidine** | **Stool consistency/frequency^†^** | **Post-intervention stool consistency/frequency^‡^** | **Post-intervention stool consistency/ frequency§** |
| --- | --- | --- | --- | --- | --- | --- | --- |
| 1. | Loose/6 per day | Loose/6 per day | Loose/6 per day | Loose/6 per day | Formed/1 per day | Formed/2 per day | Formed/1 per day |
| 2. | Loose/4 per day | Loose/4 per day | Loose/4 per day | Loose/4 per day | Formed/1 per day | Formed/1 per day | Formed/1 per day |
| 3. | Loose/6 per day | Loose/5 per day | Loose/5 per day | Loose/5 per day | Formed/2 per day | Formed/1 per day | Formed/1 per day |
| 4. | Loose/4 per day | Loose/4 per day | Loose/4 per day | Loose/4 per day | Formed/1 per day | Formed/1 per day | Formed/1 per day |
| 5. | Loose/5 per day | Loose/5 per day | Loose/5 per day | Loose/5 per day | Formed/2 per day | Formed/1 per day | Formed/1 per day |
| 6. | Loose/6 per day | Loose/6 per day | Loose/6 per day | Loose/6 per day | Formed/1 per day | Formed/1 per day | Formed/1 per day |
| 7. | Loose/7 per day | Loose/6 per day | Loose/6 per day | Loose/6 per day | Formed/1 per day | Formed/2 per day | Formed/1 per day |
| 8. | Loose/6 per day | Loose/6 per day | Loose/6 per day | Loose/6 per day | Formed/2 per day | Formed/1 per day | Formed/1 per day |
| 9 | Loose/5 per day | Loose/4 per day | Loose/4 per day | Loose/4 per day | Formed/1 per day | Formed/1 per day | Formed/1 per day |
| 10. | Loose/4 per day | Loose/4 per day | Loose/4 per day | Loose/4 per day | Formed/1 per day | Formed/1 per day | Formed/1 per day |

**^*^5^th^ day of intervention. ^†^10^th^ day of intervention. ^‡^30 days after stopping intervention with oral ranitidine. § 60 days after stopping intervention**

1. **Adverse Events:** There were no adverse events associated with this trial
