# Supplementary material for: A short course of oral ranitidine as a novel treatment for toddler’s diarrhea: a parallel-group randomized controlled trial
Source: BMC Pediatr. 2020 Aug 11;20:380. doi: 10.1186/s12887-020-02267-7 (PMC7422520; doi:10.1186/s12887-020-02267-7)
Supplement: Supplementary file 3 — Additional file 3. [file 12887_2020_2267_MOESM3_ESM.docx]

**Appendix 2 (PROFORMA)**

**STUDY TOPIC-** A short course of oral ranitidine as a novel treatment for toddler’s diarrhea: a parallel-group randomized controlled trial

**PARTICIPANT’S INFORMATION SHEET**

**STUDY CENTRE**………………………………………………………………..

**PARTICIPANT’S IDENTIFICATION NUMBER**………………………………….

**TICK OR SHADE THE APPROPRIATE OPTION:**

1. RANITIDINE GROUP [ ] C. PLACEBO GROUP [ ]
2. PROBIOTIC GROUP [ ]
3. **BIODATA:**

(a) NAME (abbreviate)……………………

(b) AGE (as at last birthday)…………….

1. GENDER: (i) MALE [ ] (ii) FEMALE [ ]
2. SOCIO-ECONOMIC CLASS (based on parents’ educational status and occupation)……………………….
3. CONTACT ADDRESS………………………………………………………………
4. CARE-GIVER’S PHONE NO…………………………………………………..
5. DAY CARE ATTENDANCE: YES [ ] NO [ ]
6. SCHOOL ATTENDANCE: YES [ ] NO [ ]
7. **DIETARY HISTORY:** (a) PICKY EATER? YES [ ] NO [ ]

(b) RATE ARTIFICIAL JUICE CONSUMPTION

(i) RARELY/NEVER [ ]

(ii) SOMETIMES [ ]

(iii) FREQUENT [ ]

(c) RATE DAILY WATER CONSUMPTION

(i) POOR [ ]

(ii) MODERATE [ ]

(iii) HEAVY [ ]

(d) RATE CONSUMPTION OF FAMILY MENU

(i) POOR [ ]

(ii) FAIR [ ]

(iii) GOOD [ ]

1. **ANTHROPOMETRY:**
2. WEIGHT (kg)…………………………………….
3. MID-ARM CIRCUMFERENCE (cm)……………………………….
4. HEIGHT (cm)………………………………………………
5. **LABORATORY DATA:**
6. STOOL ANALYSIS RESULT……………………………………………………….
7. STOOL MICROSCOPY RESULT……………………………………………………
8. STOOL CULTURE RESULT………………………………………………………..
9. **VITAL SIGNS/ STATE OF HYDRATION:**
10. TEMPRATURE (degree Centigrade)………………………………………..
11. PULSE RATE…………………………………………………..
12. RESPIRATORY RATE……………………………………………………
13. DEHYDRATION: YES [ ] NO [ ]
14. **INTERVENTION:**
15. ORAL RANITIDINE TABLETS (3 mg/kg/daily). DURATION OF TREATMENT: 10 DAYS

COMPLIANCE: (i) FULL [ ] (ii) PARTIAL [ ] (iii) DEFAULTED [ ]

1. ORAL PROBIOTICS (5 to 10 billion colony-forming units per day). DURATION OF TREATMENT: 10 DAYS

COMPLIANCE: (i) FULL [ ] (ii) PARTIAL [ ] (iii) DEFAULTED [ ]

1. ORAL PLABEBO (50 mg/day of vitamin C tablet)

DURATION OF TREATMENT: 10 DAYS

COMPLIANCE: (i) FULL [ ] (ii) PARTIAL [ ] (iii) DEFAULTED [ ]

1. **ADVERSE DRUG REACTIONS** (reported by care-giver):
2. YES [ ]
3. NO [ ]
4. IF YES, LIST:……………………………………………………………………………………………………………………………………………………………………………………………………………………………………………………………………………………………………………………………………………………………………………………………………………………………………………………………………………………………………………………………………………………………………………………………………………………………………………………
5. INDICATE DAYS OF EVENT:……………………………………………………………………………………………………………………………………………………………………………………………………………………………………………………………………………………………………………………………………………………………..
6. **PRIMARY OUTCOME MEASURES:**
7. STOOL FREQUENCY

| STUDY GROUP | PRE-TREATMENT STOOL FREQUENCY | DURING TREATMENT STOOL FREQUENCY (DAY 1) | DURING TREATMENT STOOL FREQUENCY  (DAY 5) | DURING TREATMENT STOOL FREQUENCY  (DAY 10) | STOOL  FREQUENCY  (DAY 30) |
| --- | --- | --- | --- | --- | --- |
| ORAL RANITIDINE GROUP |  |  |  |  |  |
| ORAL PROBIOTIC GROUP |  |  |  |  |  |
| ORAL PLACEBO GROUP |  |  |  |  |  |

1. STOOL CONSISTENCY

| STUDY GROUP | PRE-TREATMENT STOOL CONSISTENCY | DURING TREATMENT  DAY 1 | DITTO  DAY 5 | DITTO  DAY 10 | DITTO  DAY 30 |
| --- | --- | --- | --- | --- | --- |
| ORAL RANITIDINE GROUP |  |  |  |  |  |
| ORAL PROBIOTICS GROUP |  |  |  |  |  |
| ORAL PLACEBO GROUP |  |  |  |  |  |

1. **FOLLOW-UP AT DAY 60 (SUMMARY OF OUTCOME MEASURES):**
2. STOOL FREQUENCY PER DAY…………………………………….
3. STOOL CONSISTENCY: (i) FORMED [ ]

(ii) SEMI-FORMED [ ]

(iii) LOOSE/ WATERY [ ]

**NAME OF INVESTIGATOR**……………………………………………………………

DATE…………………………………………………………………………..
